# Characterization of Bacterial Communities in Air and Bedding Materials of Intensive Donkey Farms During Summer

**DOI:** 10.3390/microorganisms14010053

**Published:** 2025-12-26

**Authors:** Wenxuan Si, Jianpeng Zhang, Yu Zhang, Yanfei Ji, Muhammad Zahoor Khan, Yinze Chen, Zhouzhou Cheng, Jinguang Zhuang, Xia Zhao, Wenqiang Liu

**Affiliations:** 1 College of Agriculture and Biology, Liaocheng University, 1 Hunan Road, Liaocheng 252000, Chinazahoorkhan@lcu.edu.cn (M.Z.K.);; 2 Dong’e County Animal Husbandry Bureau, 99 Shuguang Street, Dong’e County, Liaocheng 252200, China

**Keywords:** donkey farms, bacterial communities, 16S rRNA sequencing, microbial aerosols, biosecurity, *Rhodococcus equi*

## Abstract

This study investigated the bacterial community composition and diversity in air and exercise yard bedding samples from large-scale donkey farms in Liaocheng, China, during summer using 16S rRNA high-throughput sequencing. Air samples were collected from five functional areas of donkey barns, while bedding samples were obtained from eight farms housing Dezhou donkeys. Sequencing analysis revealed 894 operational taxonomic units (OTUs) in air samples and 3127 OTUs in bedding samples. Alpha diversity indices indicated that the mare barn exhibited the highest microbial diversity in air, while the foal barn showed the lowest. *Actinobacteriota*, *Proteobacteria*, and *Firmicutes* were the dominant phyla across different functional areas. *Rhodococcus* was identified as the predominant airborne genus, representing a potential pneumonia risk in foals. In bedding materials, *Firmicutes*, *Actinobacteriota*, and *Proteobacteria* predominated, with *Corynebacterium*, *Salinicoccus*, and *Solibacillus* as dominant genera. Several potentially pathogenic bacteria were detected, including *Rhodococcus*, *Corynebacterium*, *Clostridium*, *Streptococcus*, and *Escherichia-Shigella.* These findings provide critical insights into the microbial ecology of intensive donkey farming environments and offer scientific evidence for developing targeted biosecurity strategies to safeguard animal health and promote sustainable livestock production.

## 1. Introduction

The donkey industry represents a regionally significant component of China’s animal husbandry sector, providing meat, milk, and hides—the latter serving as a key ingredient in traditional medicine—while also contributing to rural economic development. Liaocheng, widely recognized as the ‘Donkey Capital of China,’ serves as the core production area for the Dezhou donkey breed, underscoring the strategic importance of local farm health and biosecurity in sustaining both regional growth and the national donkey industry. In recent years, the transition from traditional smallholder farming systems to large-scale intensive operations has substantially improved production efficiency while concurrently elevating disease outbreak risks [1]. Livestock production environments are increasingly recognized as reservoirs harboring diverse microbial communities, the composition of which may be modulated by antibiotic residues and prevailing farming practices [2]. Intensive cultivation systems are characterized by high stocking densities, suboptimal environmental regulation, and inadequate manure management protocols, which collectively increase physiological stress, compromise ventilation efficacy, and facilitate waste accumulation—factors that substantially elevate infectious disease emergence risk [3,4,5].

Environmental parameters exert considerable influence on microbial community dynamics within livestock facilities. International investigations have showed that barn architectural design and ventilation efficiency significantly modulate environmental microbial assemblages [6]. Bedding substrate selection represents another critical determinant of microbial diversity, with organic materials typically supporting more complex microbial consortia, whereas scientifically optimized ventilation systems can alter airborne bacterial composition and mitigate respiratory pathogen transmission risks [7]. Furthermore, seasonal variation directly impacts pathogen prevalence and microbial community structure [8]. Notably, summer conditions promote the proliferation of opportunistic and spore-forming bacterial populations, thereby augmenting disease outbreak risks in intensive animal production systems [9].

Microbial aerosols constitute a critical biosecurity concern in contemporary livestock environments [10]. Airborne pathogens suspended in the atmosphere can be inhaled by animals, precipitating respiratory infections, diminished productivity, and compromised immune function [11]. Evidence indicates that airborne microbial diversity exhibits strong associations with prevailing environmental conditions [12]. The selection and management of bedding materials utilized in housing facilities and exercise yards directly influence air quality parameters, microbial burden, pathogen viability, and the overall resilience of farm-level microbial communities [13]. Microbial aerosol contamination is ubiquitous in intensive livestock operations, including cattle, swine, and sheep production facilities, posing substantial threats to animal health [14]. Manure, as a primary bioaerosol source, significantly elevates environmental pathogen loads when concentrations increase, thereby threatening livestock welfare while posing potential occupational hazards to farm personnel and risks to surrounding communities [15].

Previous studies have demonstrated that seasonal temperature fluctuations significantly influence the composition and diversity of microbial communities in livestock environments, with summer conditions often associated with higher microbial loads and elevated pathogen risks [16]. In Liaocheng, average temperatures are approximately 18 °C in spring and autumn, 0 °C in winter, and 25 °C in summer. Given that bacterial metabolic activity peaks during summer, we selected this season for sampling to more effectively assess biosecurity risks in intensive donkey farms. Considering the substantial economic importance of indigenous breeds such as the Dezhou donkey and the accelerating intensification of production systems, the development of evidence-based biosecurity strategies has become imperative [17]. Accordingly, this study employed 16S rRNA gene high-throughput sequencing to analyze bacterial community composition in air and exercise yard bedding samples collected during summer from eight large-scale donkey farms in Liaocheng, China. Microbial community characteristics were compared across geographical locations to provide a scientific basis for formulating targeted biosecurity interventions, safeguarding donkey health, and promoting sustainable livestock production practices. To our knowledge, this is the first investigation of air- and bedding-associated bacterial communities in intensive donkey farms during summer, characterizing microbial diversity and the distribution of potentially pathogenic genera. These findings address an important gap in livestock microbial ecology and establish an evidence-based framework for biosecurity management in intensive donkey farming systems.

## 2. Materials and Methods

### 2.1. Sample Collection

#### 2.1.1. Air Sample Collection

**Figure 1:**
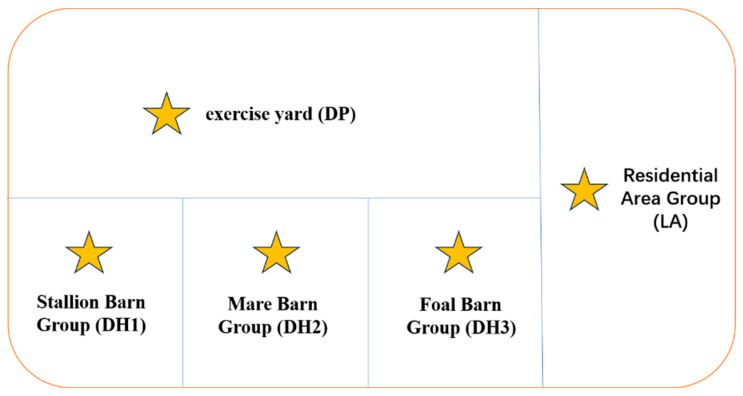
Schematic diagram of sampling locations and distribution of functional areas within donkey housing facilities.

Airborne bacterial samples were collected from five designated functional areas (Figure 1) using a six-stage Andersen cascade impactor (model BY-300, Qingdao Kaiyue Environmental Protection Equipment Co., Ltd., Qingdao, Shandong Province, China). Three independent replicate samples were collected at a standardized height of 1 m above ground level at each sampling location. Sterile 90 mm glass Petri dishes (Maisinuo, Nantong, China) containing nutrient agar medium were positioned beneath each impactor stage. Air was drawn through the system at a calibrated flow rate of 28.3 L/min for a duration of 20 min [18]. To minimize potential contamination, sampling personnel maintained an appropriate distance from the collection apparatus during operation. Following collection, individual plates were aseptically transferred into sterile sampling bags (model CYD001-1, Qingdao Haibo Biotechnology Co., Ltd., Qingdao, Shandong Province, China), sealed, transported to the laboratory under controlled conditions in a sampling box [19], and incubated at 37 ± 2 °C for 48 h. Sterile Petri dishes without air exposure were included as negative controls and subjected to the same handling, transport, and incubation conditions as the exposed samples to monitor potential contamination during sampling and laboratory processing. No technical or biological replicates were included beyond the three independent samples collected at each location.

#### 2.1.2. Bedding Sample Collection

Bedding samples were obtained from exercise yards at eight large-scale donkey production facilities (designated SO1–SO8) distributed across four counties/districts in Liaocheng, Shandong Province, China. Herd sizes ranged from 300 to 600 animals, all belonging to the Dezhou donkey breed. Approximately 15 g of surface bedding material was collected from each of three randomly selected points within each exercise yard using sterile disposable scoops (ZOLG®, Qingdao, China). Samples from individual yards were composited, transferred into sterile 50 mL nuclease-free centrifuge tubes, appropriately labeled, and maintained on dry ice until laboratory processing.

### 2.2. Laboratory Methods

#### 2.2.1. Sample Processing

For airborne samples, triplicate collections were obtained from each of the five functional areas (DP, DH1, DH2, DH3, LA). Bacterial colonies from individual agar plates were aseptically harvested using sterile inoculating loops (NEST, Wuxi, China) and transferred into sterile, nuclease-free 15 mL tubes containing phosphate-buffered saline (PBS). Suspensions were subjected to vigorous vortexing to achieve uniform bacterial cell dispersion and resuspension.

Bedding samples were processed by suspending composited material from each exercise yard in PBS within sterile, nuclease-free 15 mL tubes for subsequent downstream analyses.

#### 2.2.2. DNA Extraction

Total genomic DNA was extracted using the E.Z.N.A.^®^ Soil DNA Kit (Omega Bio-tek, Norcross, GA, USA) following the manufacturer’s protocol. DNA integrity was verified by 1% agarose gel electrophoresis, while concentration and purity were quantified using a NanoDrop spectrophotometer (NanoDrop One, Thermo Fisher Scientific, Waltham, MA, USA).

#### 2.2.3. PCR Amplification

The V3–V4 hypervariable regions of the bacterial 16S rRNA gene were amplified using barcoded universal primers 338F (5′-ACTCCTACGGGAGGCAGCAG-3′) and 806R (5′-GGACTACHVGGGTWTCTAAT-3′) [20]. Each 20 µL PCR reaction comprised 0.8 µL of each primer, 4 µL of 5× TransStart FastPfu buffer (TransGen Biotech, Beijing, China), 0.4 µL of FastPfu DNA polymerase (TransStart®, TransGen Biotech Co., Ltd., Beijing, China), 2 µL of dNTP mixture (Beyotime Biotechnology, Shanghai, China), 10 ng of template DNA (genomic DNA extracted in the previous step), and nuclease-free water (Servicebio, Wuhan, China) to final volume.

Thermal cycling parameters consisted of initial denaturation at 95 °C for 3 min; 30 cycles of denaturation at 95 °C for 30 s, annealing at 55 °C for 30 s, and extension at 72 °C for 30 s; final extension at 72 °C for 10 min; and a hold at 4 °C. Triplicate amplifications were performed for each sample. PCR products were pooled, purified using a Gel Extraction Kit (AxyPrep DNA, Axygen Biosciences, Union City, CA, USA), and quality-assessed by 2% agarose gel electrophoresis (HyAgarose™, HydraGene Biotechnology Co., Ltd., Xiamen, China).

#### 2.2.4. High-Throughput Sequencing

Sequencing libraries were constructed using the NEXTflex™ Rapid DNA-Seq Kit (Bioo Scientific, Austin, TX, USA) and sequenced on the Illumina MiSeq PE300 platform (Illumina, San Diego, CA, USA).

#### 2.2.5. Bioinformatics Analysis

Raw sequencing reads were subjected to quality filtration using fastp (https://github.com/OpenGene/fastp, v0.19.6; accessed on 1 March 2023) and merged using FLASH (http://www.cbcb.umd.edu/software/flash, v1.2.11; accessed on 1 March 2023). The OTUs were delineated at 97% sequence similarity threshold using UPARSE (http://drive5.com/uparse/, v7.1; accessed on 1 March 2023). Taxonomic assignments were performed using the RDP Classifier (http://rdp.cme.msu.edu/, v2.11; accessed on 1 March 2023) with reference to the SILVA 16S rRNA database (v138). Functional profiling was conducted using PICRUSt2 (v2.2.0) [7].

#### 2.2.6. Statistical Analysis

Comprehensive data analyses were executed on the Majorbio Cloud Platform (https://cloud.majorbio.com/; accessed on 3 March 2023). Alpha diversity indices were calculated using mothur (v1.48.0) to evaluate community richness and evenness, assess intergroup differences, and examine microbial community structural similarity among samples [9]. Linear discriminant analysis effect size (LEfSe) [21] was implemented to identify bacterial taxa exhibiting significantly differential relative abundances across groups, spanning taxonomic levels from phylum to genus.

## 3. Results

### 3.1. Airborne Bacterial Communities in Different Functional Areas During Summer

#### 3.1.1. Sequencing Performance and Data Quality

**Figure 2:**
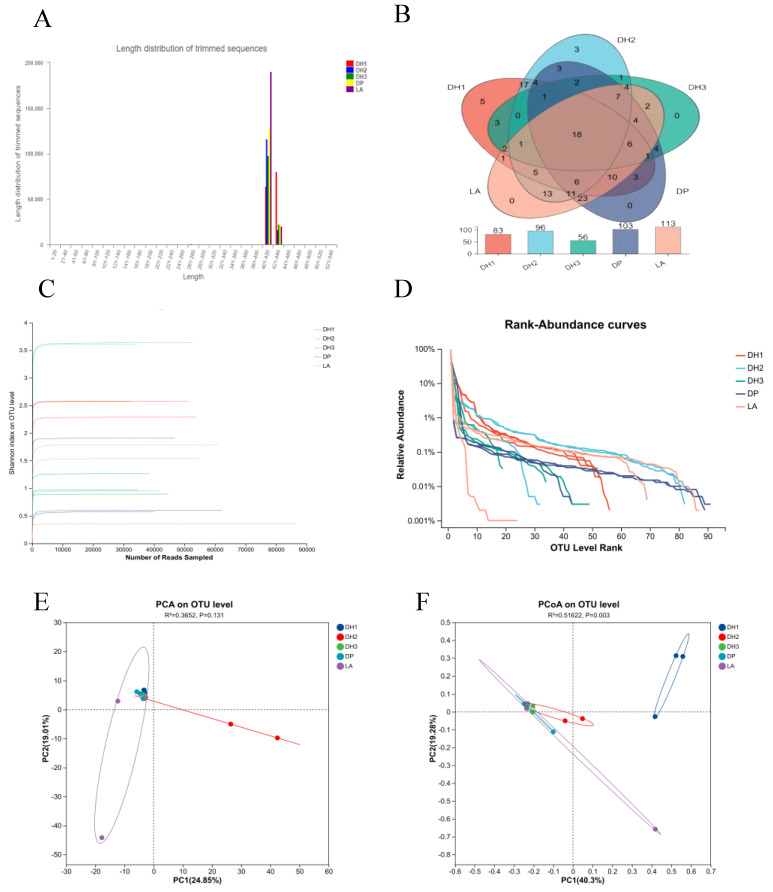
Microbial community analysis of airborne bacteria across different functional zones during summer. (**A**) Length distribution of trimmed 16S rRNA sequences from each region. (**B**) Venn diagram and bar chart showing shared and unique OTUs among air samples from different zones. (**C**) Rarefaction curves based on the Shannon diversity index, illustrating sequencing depth and diversity saturation. (**D**) Rank-abundance curves depicting OTU richness and evenness across sample groups. (**E**) Principal Component Analysis (PCA) at the OTU level, visualizing community structure and group clustering. (**F**) Principal Coordinates Analysis (PCoA) based on Bray–Curtis distance, with PERMANOVA results (R^2^ = 0.5162, *p* = 0.03) indicating significant differences among groups.

High-throughput 16S rRNA gene sequencing yielded 760,349 high-quality sequences with an average read length of 411 bp (Figure 2A). The sequence distribution across sampling locations was as follows: stallion barn (DH1) with 144,780 sequences, mare barn (DH2) with 131,884 sequences, foal barn (DH3) with 120,169 sequences, exercise yard (DP) with 151,408 sequences, and living area (LA) with 210,912 sequences.

#### 3.1.2. Operational Taxonomic Unit (OTU) Analysis and Diversity Assessment

Clustering analysis at 97% sequence similarity identified 894 operational taxonomic units (OTUs) across all samples (Figure 2B). The distribution of OTUs varied considerably among functional areas: DH1 contained 83 OTUs, DH2 had 96 OTUs, DH3 had 56 OTUs, DP had 103 OTUs, and LA had 113 OTUs. Site-specific OTUs were detected in the stallion barn (n = 5) and mare barn (n = 3), while the foal barn, exercise yard, and living area showed no unique OTUs. A core microbiome of 18 OTUs was shared among all five functional areas.

Shannon rarefaction curves showed that sequencing depth was enough for all samples, with curves approaching asymptotic values (Figure 2C). Bacterial diversity was highest in the mare barn (DH2), followed by the stallion barn (DH1), foal barn (DH3), exercise yard (DP), and living area (LA). Rank-abundance curves revealed distinct community structure patterns (Figure 2D). The exercise yard, mare barn, and living area exhibited smoother vertical profiles compared with the stallion and foal barns, indicating greater species evenness. The broader horizontal distribution observed in DH1, DH2, DP, and LA suggested higher species richness relative to DH3.

#### 3.1.3. Alpha and Beta Diversity Patterns

Alpha diversity indices varied among functional areas (Table 1). The mare barn (DH2) exhibited the highest bacterial diversity, with ACE (206.56), Chao1 (201.86), Shannon (2.73), and Simpson (0.35) indices representing the maximum values observed, while the foal barn (DH3) displayed the lowest diversity across all indices. Good’s coverage values exceeded 0.99 for all sampling sites, indicating that more than 99% of the bacterial diversity was adequately captured.

Beta diversity was further assessed using principal component analysis (PCA) and principal coordinates analysis (PCoA) (Figure 2E,F). PCA revealed that the first two principal components explained 43.89% of the total variance (PC1: 24.85%; PC2: 19.04%), with R^2^ = 0.3652 and *p* = 0.131, indicating no significant separation among groups. In contrast, PCoA showed that the first two coordinates accounted for 59.56% of the variation (PCoA1: 40.30%; PCoA2: 19.26%), with R^2^ = 0.5162 and *p* = 0.003, suggesting significant differences in microbial community composition among functional areas. Samples from the same functional areas tended to cluster together, reflecting similar bacterial community structures within each location type.

#### 3.1.4. Taxonomic Composition and Distribution

**Figure 3:**
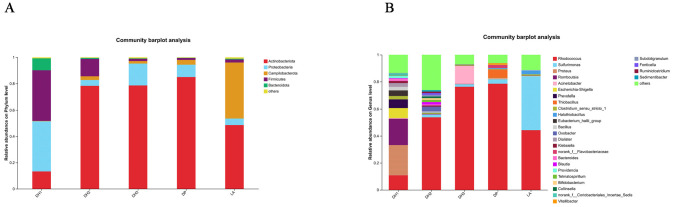
Phylum- and genus-level composition of bacterial communities in different regions during summer. (**A**) Composition of phylum levels in different regions in summer. (**B**) Composition of genus levels in different regions in summer.

The airborne bacterial communities across all functional areas were taxonomically diverse, comprising 10 phyla, 20 classes, 48 orders, 83 families, 130 genera, and 154 species. Phylum-level analysis (Figure 3A; Table 2) revealed four dominant bacterial phyla: *Actinobacteriota*, *Proteobacteria*, *Campilobacterota*, and *Firmicutes. Actinobacteriota* predominated in the mare barn (78.30%), foal barn (78.58%), and exercise yard (85.00%). In contrast, the stallion barn exhibited a more balanced distribution, with *Proteobacteria* (38.22%) and *Firmicutes* (38.38%) as co-dominant phyla. The living area showed a mixed profile dominated by *Actinobacteriota* (48.64%) and *Campilobacterota* (42.44%).

Genus-level analysis (Figure 3B; Table 3) identified *Rhodococcus* as the predominant genus in the mare barn (73.47%), foal barn (78.25%), and exercise yard (84.41%). The stallion barn displayed a more diverse genus composition, with *Proteus* (24.62%), *Acinetobacter* (21.50%), and *Rhodococcus* (11.91%) as dominant taxa. The living area was characterized by *Sulfurimonas* (42.28%) and *Rhodococcus* (47.07%) as the primary genera.

### 3.2. Bacterial Communities in Exercise Yard Bedding Materials

#### 3.2.1. Sequencing Data and Quality Assessment

**Figure 4:**
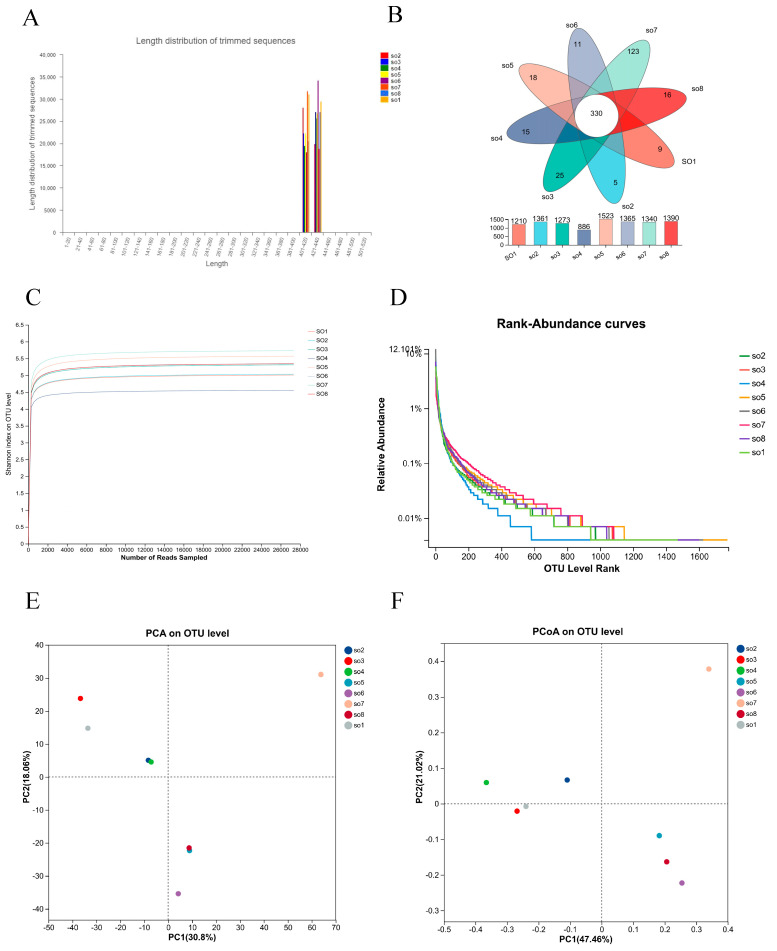
Analysis of bacterial communities in exercise yard bedding. (**A**) Sequencing length distribution diagram of the bedding sample, showing read length profiles. (**B**) Comparison of OTU numbers among bacterial samples from different groups. (**C**) Shannon diversity curves illustrating sequencing depth and community diversity. (**D**) Rank-abundance curves displaying species richness and evenness. (**E**) Principal component analysis (PCA) at the OTU level, showing variance explained by PC1 and PC2. (**F**) Principal coordinates analysis (PCoA) at the OTU level, illustrating clustering patterns of microbial communities across groups.

Analysis of bedding samples generated 404,109 high-quality sequences with an average read length of 418 bp (Figure 4A), demonstrating excellent sequencing quality across all farm locations.

#### 3.2.2. OTU Diversity and Community Structure

Clustering analysis identified 3127 OTUs at 97% similarity threshold (Figure 4B), indicating substantially higher bacterial diversity in bedding materials compared with airborne communities. A core microbiome of 330 OTUs was shared among all farms. Farm-specific OTUs were distributed as follows: SO1 (n = 9), SO2 (n = 5), SO3 (n = 25), SO4 (n = 15), SO5 (n = 18), SO6 (n = 11), SO7 (n = 123), and SO8 (n = 16). Shannon rarefaction curves reached saturation for all farms, confirming attempt sequencing depth (Figure 4C). Rank-abundance curves exhibited broad horizontal spans with smooth vertical declines, indicating high species richness and evenness across all bedding samples (Figure 4D).

#### 3.2.3. Alpha and Beta Diversity Analysis

Alpha diversity metrics varied among farms (Table 4), with SO5 exhibiting the highest species richness (ACE: 2482.95; Chao1: 2491.67) and SO4 showing the lowest (ACE: 1709.84; Chao1: 1496.09). SO7 showed the highest Shannon diversity index (5.73), while SO4 had the lowest (4.55). Simpson indices were consistently low across all farms (0.011–0.027), indicating high evenness. Beta diversity analysis through PCA and PCoA revealed similar patterns to the airborne communities (Figure 4E,F). PCA explained 48.23% of the total variance (PC1: 30.8%, PC2: 18.06%), while PCoA accounted for 68.58% (PCoA1: 47.46%, PCoA2: 21.02%). The ordination patterns indicated relatively similar bacterial community structures among different farms.

#### 3.2.4. Taxonomic Diversity and Composition

**Figure 5:**
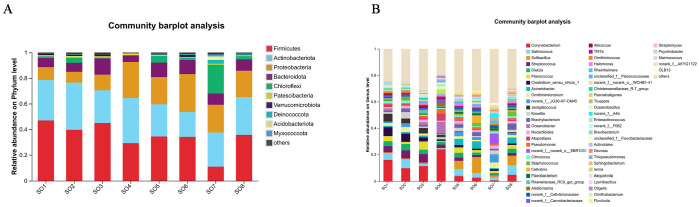
Relative abundance of bacterial phyla and genera in bedding samples. (**A**) Relative abundance of phylum level. (**B**) Relative abundance of genus level.

Bedding bacterial communities exhibited remarkable taxonomic diversity, encompassing 33 phyla, 75 classes, 192 orders, 373 families, 806 genera, and 1337 species. Phylum-level composition (Figure 5A; Table 5) was dominated by three major phyla across all farms: *Firmicutes*, *Actinobacteriota*, and *Proteobacteria*, although their relative abundances varied considerably. *Firmicutes* was the predominant phylum in six farms (SO1, SO2, SO3, SO5, SO6, and SO8), with abundances ranging from 34.02% to 46.27%. *Actinobacteriota* dominated in SO4 (35.56%) and SO7 (26.65%), while showing substantial presence across all other farms.

Genus-level analysis (Figure 5B; Table 6) identified ten dominant genera: *Corynebacterium*, *Salinicoccus*, *Solibacillus*, *Streptococcus*, *Dietzia*, *Planococcus*, *Clostridium sensu stricto 1*, *Acinetobacter*, *Ornithinimicrobium*, and *norank_f__JG30-KF-CM45*. *Corynebacterium* showed the highest overall abundance, particularly in SO4 (23.88%) and SO1 (15.91%). *Salinicoccus* was consistently present across farms, with notable abundance in SO8 (7.36%) and SO2 (6.73%). *Solibacillus* exhibited considerable variation among farms, ranging from 0.24% in SO4 to 12.09% in SO6.

#### 3.2.5. Functional Prediction Analysis

**Figure 6:**
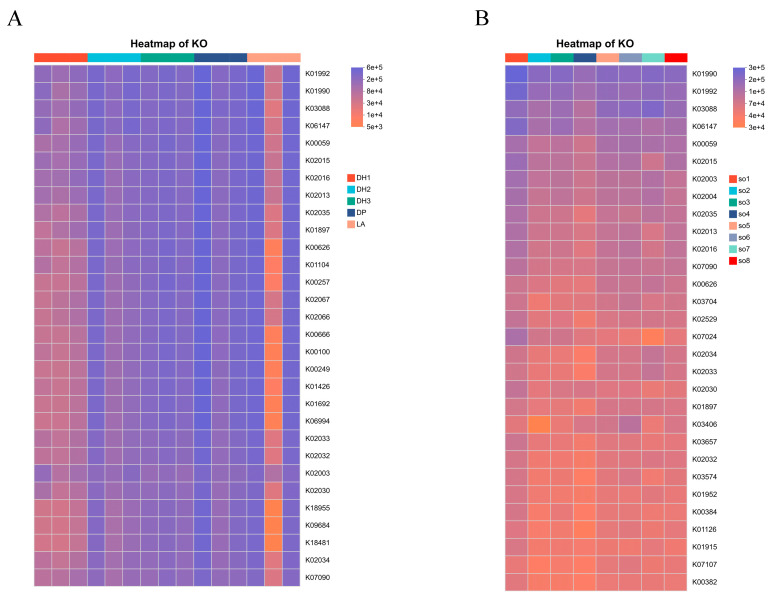
Functional prediction heatmaps (KEGG KO level). (**A**) Predicted functional profiles of airborne bacterial communities across different functional zones, showing the relative abundance of major KEGG KOs. Colors indicate different zones (DH1, DH2, DH3, DP, LA). (**B**) Predicted functional profiles of bedding bacterial communities from eight donkey farms, showing the relative abundance of major KEGG KOs. Colors indicate different farms (SO1–SO8).

Heatmaps of KEGG KO functional predictions generated by PICRUSt2 are presented in Figure 6A,B. Air samples across different functional zones exhibited distinct distributions of metabolism-related KOs, while bedding samples from different farms displayed variation in functional abundances. The major predicted functional categories included carbohydrate metabolism, amino acid metabolism, energy metabolism, as well as KOs associated with antibiotic resistance and virulence.

## 4. Discussion

The composition and dynamics of environmental microbial communities are linked to disease occurrence and transmission in intensive livestock systems. In this study, we used high-throughput sequencing to investigate bacterial community composition and abundance in air and bedding materials across five functional areas and eight donkey farms during summer in Liaocheng, China. Our findings revealed distinct microbial structures and assessed the pathogenic potential of dominant taxa, providing a foundation for biosecurity management in intensive donkey farming.

Microbial aerosols act as vectors for pathogen transmission, facilitating disease spread within and between housing facilities [22]. Potentially pathogenic genera pose threats to animal health and production [8]. Sequencing results showed the mare barn had the highest alpha diversity and richness, likely reflecting stocking density, animal activity, and organic matter accumulation, whereas the foal barn displayed lower diversity and richness, suggesting superior air quality due to smaller body size, lower occupancy, and more frequent cleaning protocols for young animals.

Examining the taxonomic composition of these airborne communities revealed important patterns with implications for disease risk. At the phylum level, *Actinobacteriota* emerged as the predominant bacterial group across all functional areas, consistent with previous findings that identified *Actinobacteriota* as the dominant phylum in aerosol [23]. However, notable variations in community composition were observed among different functional areas. In the stallion barn (DH1), *Firmicutes* (38.38%) and *Proteobacteria* (38.22%) were co-dominant phyla, indicating a balanced distribution rather than a single predominance, whereas the residential area (LA) was characterized by *Actinobacteriota* (48.64%) and *Campilobacterota* (42.44%) as the dominant phyla. At the genus level, *Proteus* was identified as the predominant genus in the stallion barn, whereas *Rhodococcus* dominated in all other functional areas. This widespread prevalence of *Rhodococcus* suggests that this genus plays a pivotal role in shaping airborne microbial community dynamics within donkey farm environments.

It should be noted that 16S rRNA gene sequencing of the V3–V4 region provides genus-level resolution only and cannot distinguish pathogenic from non-pathogenic species. Within this limitation, the dominance of *Rhodococcus* in our study raises significant biosecurity concerns, as this genus includes *R. equi*, the primary causative agent of suppurative bronchopneumonia in foals [24,25]. Although clinical cases of *R. equi* pneumonia in donkeys have not been documented in China, the intensification of donkey farming operations raises concerns regarding potential aerosol-mediated transmission. Bedding materials also represent a critical component of microbial ecology, functioning both as reservoirs for potential pathogens and as modulators of airborne microbial concentrations [14,26,27]. Our analysis revealed consistent dominance of *Firmicutes*, *Actinobacteriota*, and *Proteobacteria* across farms, in line with previous findings in cattle farm bedding [28]. At the genus level, *Corynebacterium*, *Salinicoccus*, and *Solibacillus* were most abundant. While *Solibacillus* species are generally considered beneficial due to their antifungal and antibacterial metabolites [29], the persistent occurrence of Corynebacterium warrants caution, as pathogenic members such as *C. pseudotuberculosis* are well-documented causes of caseous lymphadenitis in small ruminants [30]. In addition, *Clostridium* exceeded 1% relative abundance in all exercise yard bedding samples, with toxigenic *strains* known to cause enteric diseases in livestock [31]; *Streptococcus* and *Acinetobacter* also surpassed 1% in multiple farms, and pathogenic species within these genera are associated with diarrhea and suppurative infections in livestock [32,33]. Overall, these findings highlight both risks and ecological functions of airborne and bedding-associated microbiota, emphasizing the need for vigilant monitoring and targeted biosecurity measures in intensive donkey farming. Future studies should incorporate confirmatory assays, such as qPCR targeting the *vapA* virulence gene, or complementary approaches that enable species-level resolution. In addition, functional predictions varied across different functional zones and farms. Air samples from the mare barn exhibited elevated levels of virulence-associated functions compared to other areas, likely reflecting high stocking density and organic matter accumulation, whereas the stallion barn showed higher abundances of energy metabolism functions, suggesting distinct environmental influences on microbial functional profiles. For bedding samples, certain farms (e.g., SO3 and SO5) displayed higher abundances of antibiotic resistance-related functions than others, potentially reflecting differences in antibiotic usage or management practices. These spatial variations highlight the need for tailored biosecurity measures that account for functional differences among zones and farms.

The integration of our findings from both airborne and bedding microbial communities reveals a complex biosecurity landscape in intensive donkey farming. The predominance of *Rhodococcus* in air samples across multiple functional areas, combined with the consistent presence of pathogenic genera such as *Corynebacterium*, *Clostridium*, *Streptococcus*, and *Acinetobacter* in bedding materials, indicates that both transmission routes merit equal attention in disease prevention strategies. We acknowledge that the use of the Andersen impactor on nutrient agar represents a culture-dependent approach, inherently favoring fast-growing and culturable bacteria. Therefore, our results reflect the culturable fraction of airborne microbiota rather than an unbiased overview of the entire community. Given that summer conditions may intensify bacterial proliferation through elevated temperatures and humidity, seasonal management protocols should be prioritized during this critical period. Based on our findings, we recommend implementing a multi-faceted biosecurity approach that includes strengthening environmental disinfection protocols with emphasis on regular aerosol disinfection conducted at least weekly, ensuring adequate personnel protection through good ventilation and the use of personal protective equipment such as masks and gloves, implementing bedding material pretreatment procedures before introduction to animal housing areas, establishing routine monitoring of bedding moisture levels to prevent conditions conducive to bacterial growth, and developing protocols for timely partial or complete bedding replacement based on aim assessment criteria. In particular, frequent removal of manure and bedding in the foal barn is essential to reduce the resuspension reservoir of Rhodococcus in the air, thereby mitigating the risk of opportunistic pneumonia in young animals. These evidence-based management strategies can significantly reduce microbial load and minimize pathogen transmission risks, thereby protecting animal health and supporting productivity in large-scale donkey farming operations.

## 5. Conclusions

This study provides the first comprehensive characterization of bacterial communities in both air and bedding materials of large-scale donkey farms in China during summer, revealing significant spatial variation in microbial diversity. The findings establish critical baseline data for understanding environmental microbial ecology in intensive donkey production systems and offer practical, evidence-based guidance for implementing targeted disease prevention strategies. The functional prediction results, highlighting enhanced metabolic activity in airborne samples and enriched antibiotic resistance and virulence functions in bedding samples, further support our recommendation for a multi-faceted biosecurity strategy. By addressing both airborne and bedding-associated pathogen reservoirs through enhanced environmental management practices, the donkey industry can improve biosecurity standards, safeguard animal welfare, and advance toward more sustainable and resilient production systems.

## Figures and Tables

**Table 1:** Calculation results of Alpha diversity index in airborne samples.

Group	Ace Index	Chao Index	Shannon Index	Simpson Index	Coverage Index
Stallion Barn Group (DH1)	100.42	99.33	2.48	0.21	0.99
Mare Barn Group (DH2)	206.56	201.86	2.73	0.35	0.99
Foal Barn Group (DH3)	52.56	63.67	1.04	0.60	0.99
Exercise Yard Group (DP)	145.45	145.80	1.03	0.67	0.99
Residential Area Group (LA)	178.34	179.98	1.22	0.68	0.99

Note: Group differences were tested using the Kruskal–Wallis test. Results showed no significant differences among groups (Ace χ^2^ = 4.233, *p* = 0.375; Chao χ^2^ = 4.100, *p* = 0.393; Shannon χ^2^ = 6.833, *p* = 0.145; Simpson χ^2^ = 6.733, *p* = 0.151; Coverage χ^2^ = 5.168, *p* = 0.271). One-way ANOVA yielded similar non-significant results (Ace *p* = 0.163; Chao *p* = 0.230; Shannon *p* = 0.079; Simpson *p* = 0.093; Coverage *p* = 0.741).

**Table 2:** Relative abundance of bacterial phyla in summer air samples from different regions.

Phylum	DH1	DH2	DH3	DP	LA
*Actinobacteriota*	13.29%	78.30%	78.58%	85.00%	48.64%
*Proteobacteria*	38.22%	4.44%	16.69%	9.42%	4.97%
*Campilobacterota*	0.28%	2.82%	1.88%	3.60%	42.44%
*Firmicutes*	38.38%	13.28%	1.75%	1.50%	2.31%
*Bacteroidota*	9.08%	0.76%	0.37%	0.18%	0.61%

**Table 3:** Relative abundance of bacterial genera in summer air samples from different regions.

Genus	DH1	DH2	DH3	DP	LA
*Rhodococcus*	11.91%	73.47%	78.25%	84.41%	47.07%
*Sulfurimonas*	0.04%	2.80%	1.88%	3.56%	42.28%
*Proteus*	24.62%	0.03%	0.17%	0.58%	0.09%
*Romboutsia*	21.50%	0.27%	0.14%	0.02%	0.00%
*Acinetobacter*	0.57%	0.13%	13.73%	0.02%	0.18%
*Eschericha-Shigella*	8.10%	*	0.11%	0.25%	0.15%
*Prevotella*	6.91%	0.27%	0.18%	0.08%	0.15%
*Thiobacillus*	0.01%	*	*	6.51%	0.02%
*Clostridium sensu stricto 1*	2.72%	1.01%	0.40%	0.41%	0.86%
*Halothiobacillus*	*	0.19%	0.09%	0.71%	3.04%

Note: * indicates that the relative abundance in this group is extremely low.

**Table 4:** Calculation results of Alpha diversity index in bedding samples.

Constituencies	Ace Index	Chao Index	Shannon Index	Simpson Index	Sobs Index
SO1	1997.61	2026.93	5.00	0.02	1454
SO2	2352.97	2343.03	5.03	0.021	1590
SO3	2143.86	2175.07	5.31	0.017	1596
SO4	1709.84	1496.09	4.55	0.027	962
SO5	2482.95	2491.67	5.56	0.013	1780
SO6	2279.19	2312.38	5.34	0.023	1619
SO7	1743.07	1765.69	5.73	0.011	1436
SO8	2213.90	2297.19	5.34	0.016	1602

Note: The bedding group contained only one sample, which precluded statistical testing; only descriptive results are reported.

**Table 5:** Relative abundance of bacterial phyla in bedding samples.

Phylum	SO1	SO2	SO3	SO4	SO5	SO6	SO7	SO8
*Firmicutes*	46.27%	39.14%	44.32%	28.68%	34.02%	34.12%	10.70%	35.52%
*Actinobacteriota*	32.27%	37.55%	26.18%	35.56%	25.58%	19.71%	26.65%	29.75%
*Proteobacteria*	10.3%	8.53%	12.45%	28.43%	21.54%	29.96%	21.72%	20.75%
*Bacteroidota*	7%	6.97%	12.59%	4.94%	11.14%	10.66%	8.63%	8.73%
*Chloroflexi*	1.34%	4.05%	0.64%	0.54%	5.34%	1.87%	22.85%	2.54%
*Verrucomicrobiota*	1.02%	1.00%	0.89%	0.12%	0.33%	1.53%	0.22%	0.38%
*Deinococcota*	0.09%	0.27%	0.13%	0.19%	0.29%	0.47%	2.45%	0.56%
*Acidobacteriota*	0.02%	0.07%	0.01%	0.01%	0.09%	0.04%	2.77%	0.16%

**Table 6:** Relative abundance of genus level.

Genus	SO1	SO2	SO3	SO4	SO5	SO6	SO7	SO8
*Corynebacterium*	15.91%	9.61%	11.19%	23.88%	3.84%	2.71%	0.72%	4.61%
*Salinicoccus*	5.09%	6.73%	1.55%	0.80%	5.12%	3.25%	2.12%	7.36%
*Solibacillus*	0.65%	0.65%	1.13%	0.24%	6.26%	12.09%	1.43%	7.09%
*Streptococcus*	4.66%	6.13%	6.45%	2.61%	2.41%	1.53%	0.29%	1.99%
*Dietzia*	3.79%	4.81%	3.33%	2.17%	3.67%	1.86%	1.45%	3.34%
*Planococcus*	3.81%	1.63%	6.21%	0.64%	4.10%	1.89%	1.56%	2.83%
*Clostridium sensu stricto 1*	6.80%	6.24%	3.08%	1.01%	1.75%	1.08%	1.38%	1.12%
*Acinetobacter*	1.01%	1.14%	1.40%	0.63%	4.74%	5.59%	0.77%	4.49%
*Omithinimicrobium*	2.04%	3.86%	1.34%	0.92%	1.91%	1.02%	4.25%	1.85%
*norank_f JG30-KF-CM45*	1.18%	2.89%	0.48%	0.39%	3.24%	1.39%	5.90%	1.47%

## Data Availability

The original contributions presented in this study are included in the article. Further inquiries can be directed to the corresponding authors.
